# Effectiveness of supplementary protocols for filling material removal after sealer ultrasonic activation - a laboratory investigation

**DOI:** 10.1590/1807-3107bor-2025.vol39.034

**Published:** 2025-04-04

**Authors:** Amanda Freitas da ROSA, Dayana Mara Silva CHAVES, Luiz Carlos de Lima DIAS-JUNIOR, Gabriela Pasqualin GHIDINI, Julia Menezes SAVARIS, Rayssa Sabino da SILVA, Roberta Pinto PEREIRA, Eduardo Antunes BORTOLUZZI, Cleonice da Silveira TEIXEIRA, Lucas da Fonseca Roberti GARCIA

**Affiliations:** (a) Universidade Federal de Santa Catarina – UFSC, Health Sciences Centre, Department of Dentistry, Endodontics Division, Florianópolis, SC, Brazil.; (b) Universidade Estadual de Campinas – Unicamp, Piracicaba School of Dentistry, Department of Restorative Dentistry, Piracicaba, SP, Brazil.; (c) University of Louisville, Endodontics Division,Department of Diagnosis & Oral Health, Louisville, KY, USA.

**Keywords:** Endodontics, Root Canal Therapy, Root Canal Obturation, Root Canal Filling Materials

## Abstract

Ultrasonic activation of the endodontic sealer makes it difficult to remove the material during endodontic reintervention. Therefore, supplementary removal protocols should be tested to optimize the removal of the remaining filling material. This study assessed the effectiveness of supplementary protocols for filling material removal after sealer ultrasonic activation (UA). Sixty teeth were prepared and distributed into two groups: UA and No UA of the sealer before obturation. Teeth were re-instrumented and two supplementary removal protocols were tested, resulting in six groups (n = 10): NoUA; NoUA+XP (XP-endo Finisher); NoUA+CS (Clearsonic-R1); UA; UA+XP; and UA+CS. Root canals were analyzed under stereomicroscopy and scanning electron microscopy for quantification of the remaining filling material. Considering the total root canal area, the NoUA+CS group had the lowest remaining filling material compared to NoUA+XP, UA+XP and UA+CS groups (p < 0.05). When the root thirds were compared, there was no statistical difference among groups (p > 0.05). The XP-endo Finisher instrument demonstrated the lowest effectiveness when used as a supplementary step. In contrast, the Clearsonic-R1 insert exhibited the highest performance.

## Introduction

Ultrasonic activation of endodontic sealer increases its mechanical retention within the root canal walls.^1-3^ When the sealer is activated, it penetrates deeper into the dentinal tubules, forming tags in greater number, density, and length.^1-3^ For this reason, studies recommend ultrasonic activation of endodontic sealer during root canal obturation.^1-3^ On the other hand, ultrasonic activation hinders the removal of the filling material when non-surgical endodontic reintervention is necessary^4^after the failure of the primary endodontic treatment.^5^

Currently, several instrumentation systems are available for filling material removal.^6,7^ However, no protocol can completely remove the filling material from the root canal.^6,7^ Therefore, supplementary protocols for the removal of the remaining filling material have been proposed.^8-11^ Among these supplementary protocols, the XP-endo Finisher rotary instrument (FKG Dentaire, La Chaux-de-Fonds, Switzerland) and the Clearsonic-R1 ultrasonic insert (Helse Ultrasonic, Santa Rosa de Viterbo, SP, Brazil) are widely used.^8-11^

The XP-endo Finisher rotary instrument was developed to complement root canal cleaning after shaping.^8,9^ However, it has proven to be effective in removing the remaining filling material from the root canal walls as an additional step in endodontic reintervention.^8,9^ According to its manufacturer, this instrument is made of MaxWire nickel-titanium alloy, which makes it flexible and at the same time resistant to cyclic fatigue. In addition, it has a snake-like shape, its tip diameter is 0.25 mm, and there is no taper variation.^8,9^

When the XP-endo Finisher instrument is inserted into the root canal and exposed to body temperature, it expands, a phenomenon known as martensitic-austenitic transformation.^11^ In the martensitic phase, the instrument has a straight shape, while in the austenitic phase, it takes on a “sickle” shape in its final 10 mm of length.^8^ At this stage, the instrument acts in areas that are difficult to reach by the conventional instruments used previously.^8^

Conversely, the Clearsonic-R1 ultrasonic insert has a different mode of action than the abovementioned instrument.^11^ It promotes friction on the root canal walls, which generates heating and vibration, leading to debris removal.^11^ In addition, its arrow-shaped tip allows the insert to reach untouched areas after traditional root canal instrumentation.^11^

According to da Silva Machado, et al.^4^(2021), ultrasonic activation of the endodontic sealer leads to a higher percentage of filling material residue attached to the root canal walls after non-surgical endodontic reintervention. However, no study has assessed the effectiveness of supplementary removal protocols such as the XP-endo Finisher instrument and the Clearsonic-R1 insert in root canals obturated after ultrasonic activation of the endodontic sealer.

Therefore, the purpose of this *in vitro* study was to assess the effectiveness of supplementary protocols (XP-endo Finisher and Clearsonic-R1) in removing filling material following sealer ultrasonic activation. The following null hypotheses were tested: a) there is no significant difference in the amount of filling material residue among groups obturated with and without ultrasonic activation of the sealer; b) the different supplementary protocols used for remaining filling material removal have similar results; and c) similar results are obtained in the different root canal levels (cervical, middle, and apical).

## Methods

**Figure 1:**
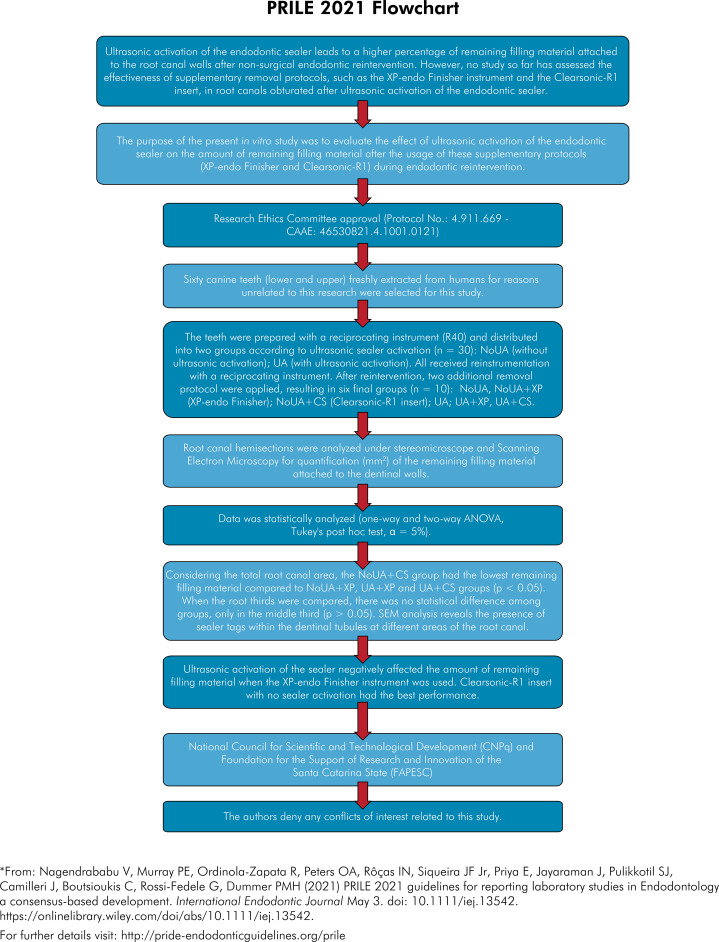
PRILE 2021 flowchart.

The manuscript of this laboratory study has been written according to 2021 Preferred Reporting Items for Laboratory studies in Endodontology (PRILE) guidelines^12^(Figure 1).

### Ethical approval

The present research was prospectively reviewed and approved by a duly constituted ethics committee (Protocol No.: 4,911,669 - CAAE: 46530821.4.1001.0121) and performed following the ethical standards laid down in the 2008 Declaration of Helsinki.

### Sample size calculation

The G*Power 3.1 program (G*Power, 3.1.7, Heinrich Heine, Universität Dusseldorf, Germany) was used to calculate the sample size. Based on previous data,^8,10^ the following parameters were considered: error α=0.05, test power (1-β) = 0.80, and effect size = 0.80. The type of power analysis was set a priori (compute required sample size - given α, power, and effect size) using ANOVA (fixed effects, special, main effects and interactions). Therefore, the sample size calculation showed that ten specimens per group were sufficient for the proper application of statistical analysis.

### Specimen selection

Sixty freshly extracted canines (lower and upper) from humans were used in this study. The teeth were carefully inspected under magnification (4×). The selected teeth had a straight, fully formed root and a single canal. After radiographic examination, the teeth were homogeneously distributed according to their root canal anatomy and volume. Teeth with previous endodontic treatment, extensive carious lesions, root cracks and fractures, calcifications, and internal and external resorptions were discarded. During selection, the teeth were also standardized concerning their initial apical diameter corresponding to a size 10 K-file (Dentsply Maillefer, Ballaigues, Switzerland), and a total length of 25 mm. Teeth with a greater apical diameter were excluded from the final sampling.

After cleaning the external tooth surface with an ultrasonic insert (T1-S; Schuster Equipamentos Odontológicas, Santa Maria, Brazil), the teeth were immersed in a 0.1% thymol solution for disinfection for 48 hours. Then, the teeth were washed in running water for 24 hours, individually stored in a plastic recipient containing gauze soaked with distilled water, and kept in an oven at 37°C until starting the experiment.

The entire experiment, from tooth selection to endodontic reintervention (filling material removal, root canal re-instrumentation, and use of the supplementary protocol), was performed by a single operator.

### Root canal preparation

The teeth had their crowns removed at the cementoenamel junction with a double-sided diamond disk No. 7016 (American Burrs, Palhoça, Brazil) coupled to a straight handpiece under constant water cooling. Next, the working length (WL) was determined by introducing a size 10 K-file (Dentsply Maillefer) into the root canal until its tip was visualized in the apical foramen. Then, 1 mm was subtracted from this length.

**Figure 2:**
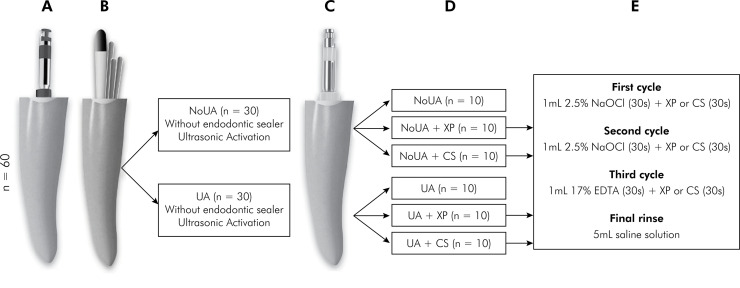
Schematic representation of the experimental design: (a) root canal preparation; (b) root canal obturation. Distribution of the specimens into the two initial groups: NoUA and UA; (c) endodontic reintervention; (d) distribution of the specimens into the six experimental groups according to the supplementary protocol used for the filling material removal; (e) the final irrigation protocol applied in the groups that used the XP-endo Finisher instrument or the Clearsonic-R1 insert as an additional step for the remaining filling material removal.

All root canals were prepared with a reciprocating instrument (R40; 40/.06) (Reciproc; VDW GmbH, Munich, Germany) coupled to the X-Smart Plus endodontic motor (Dentsply Maillefer) in the “ALL RECIPROC” mode, according to the manufacturer recommendations (Figure 2a). First, the canal was flushed with 2.5% NaOCl solution (Asfer, São Caetano do Sul, Brazil). Subsequently, the activated instrument was gradually inserted into the root canal, and after three pecking movements, it was removed from the canal for cleaning with sterile gauze soaked in 70% alcohol. At each gradual advancement and removal of the instrument for cleaning, 2 mL of 2.5% NaOCl solution was used for irrigation. The irrigation was performed with a syringe (Ultradent Products Inc., South Jordan, USA) and NaviTip 30 ga needle (Ultradent Products Inc.) inserted until 2 mm before the WL. Apical patency was checked with a size 10 K-file (Dentsply Maillefer) during the entire root canal preparation. The biomechanical preparation was considered complete when the instrument reached the WL. Each reciprocating instrument was used to prepare four root canals.

At the end of the preparation, 3 mL of 17% ethylenediaminetetraacetic acid (EDTA) (Biodinâmica, Ibiporã, Brazil) was used for 3 minutes, followed by 3 mL of 2.5% NaOCl solution for 3 minutes. Finally, 5 mL of saline solution was used to neutralize the action of the irrigating solutions.^1^ The root canal was then dried with size 40 absorbent paper points (Tanari, São Paulo, Brazil).

The prepared teeth were identified and kept in a plastic recipient containing a gauze soaked with distilled water to maintain the humidity and stored in an oven at 37°C until the root canal obturation.

### Root canal obturation

Initially, the specimens were randomly distributed (www.random.org) into two groups (n = 30) according to the ultrasonic activation of an epoxy resin-based endodontic sealer (AH Plus Jet; Dentsply Tulsa Dental Specialties, Tulsa, USA), as shown in Figure 2b.

The endodontic sealer was mixed according to the manufacturer’s recommendation. Then, a layer of wax No. 7 (Lysandra, São Paulo, Brazil) was applied to the root apex to simulate a closed system and prevent the sealer’s leakage. Before the placement of the endodontic sealer in the root canal, a master gutta-percha cone (Reciproc R40; VDW GmbH) was selected.

In the experimental groups in which the ultrasonic activation of the endodontic sealer was performed (UA groups), the sealer was placed in the root canal in circumferential movements using the master gutta-percha cone previously selected. After completely filling the root canal, the endodontic sealer was activated with an ultrasonic insert (Irrisonic; Helse Ultrasonic) coupled to a low-power (10%) ultrasonic device (Sonic Laxis BP LED; Schuster Equipamentos Odontológicos), previously calibrated. The ultrasonic insert was positioned at 2 mm from the WL and activated for 20 seconds in the buccolingual direction.^4^ Next, the master gutta-percha cone was introduced in the root canal until the WL. Then, a size 25 nickel-titanium finger spreader (Dentsply Maillefer) was slowly inserted into the root canal to laterally compact the master cone and create a space for inserting B7 accessory cones (Tanari). After the maximal placement of accessory cones, a thermoplastic tip (Easy, Belo Horizonte, Brazil) was used to cut and remove the excess filling material. Finally, a cold plugger (Odous de Deus, Belo Horizonte, Brazil) was used for the vertical compression of the cones. In the non-activated experimental groups (No UA groups), the root canal obturation was performed as previously described, with no ultrasonic activation of the endodontic sealer.

The quality of the obturation was carefully checked by periapical radiographs taken in the buccolingual and mesiodistal directions. Root canals with voids and gaps between sealer and gutta-percha cones and gaps between the filling material and root canal walls were discarded and replaced.^4^ The root canal entrance was sealed with temporary restorative cement (Cimpat; Septodont, Saint-Maur-des-Fossés, France), and the specimens were again stored in an oven, under the same conditions described above, for 30 days until complete hardening of the endodontic sealer.^4^

### Endodontic reintervention

The specimens were randomly redistributed (www.random.org) into six groups (n = 10) according to the protocol adopted for the filling material removal (Figure 2c).

All experimental groups had their filling material removed and root canals re-instrumented with the same system (Reciproc R40 and R50, respectively), following the manufacturer’s recommendations. Initially, a No. 2 Gates-Glidden drill (Dentsply Maillefer), positioned 5 mm from the WL, was used to perforate the gutta-percha in the cervical third of the root canal. The R40 (40/.06) instrument (Reciproc; VDW GmbH) was used for the filling material removal. The instrument advanced into the root canal in a pecking motion, with light apical pressure and brush movements against the root canal walls. Next, the re-instrumentation of the root canal was performed with the R50 (50/.05) instrument (Reciproc; VDW GmbH) until reaching the WL, as described above.^13^ Each reciprocating instrument was used to perform the reintervention (R40 - filling material removal and R50 - re-instrumentation) in four root canals.

The same irrigation protocol used during root canal preparation was applied in the stages of endodontic reintervention. Apical patency was obtained with a size 10 K-file (Dentsply Maillefer) on all specimens. Filling material removal was considered complete when the instrument reached the WL, when there was no evidence of filling material attached to the instrument’s cutting blades, and when no more filling material was visible in the reflux of the irrigating solution.

After using the reciprocating instruments (R40 and R50), four groups underwent a supplementary approach to remove the remaining filling material (Figure 2d). For this additional step, and based on the protocol adopted by Tavares et al.^11^(2021), the XP-endo Finisher instrument (FKG Dentaire) and the Clearsonic-R1 ultrasonic insert (Helse Ultrasonic) were used.

In the UA+XP and NoUA+XP groups, the XP-endo Finisher instrument was operated at 800 rpm and 1 Ncm torque, according to the manufacturer’s recommendations. The plastic tube surrounding the instrument was cooled using the Endo-Ice spray (Maquira Dental Products Group, Maringá, Brazil) to keep the instrument straight and allow its calibration at the WL. Then, to properly simulate a clinical situation and allow the instrument to change from the martensitic to the austenitic phase when exposed to body temperature, the irrigating solutions (2.5% NaOCl solution and 17% EDTA) were heated in a water bath to 37°C before their use. The root canal was irrigated with 1 mL of 2.5% NaOCl solution for 30 seconds, and then the instrument was inserted and used at the WL for 30 seconds (1^st^ cycle). After cleaning the instrument, the same procedure was repeated (2^nd^ cycle). Then, the root canal was irrigated with 1 mL of 17% EDTA for 30 seconds, and the XP-endo Finisher instrument was used again for 15 seconds (3^rd^ cycle).^11^ Finally, the root canal was irrigated with 5 mL of saline solution, aspirated, and dried with absorbent paper points (Tanari) (Figure 2e).

In the UA+CS and NoUA+CS groups, the ultrasonic insert was coupled to a low-power (10%) ultrasonic device (Sonic Laxis BP LED; Schuster Equipamentos Odontológicos), previously calibrated. First, the root canal was irrigated with 1 mL of 2.5% NaOCl solution for 30 seconds. Next, the insert was placed up to the WL and activated for 30 seconds in movements against the root canal walls (1^st^ cycle). After cleaning the insert, the same procedure was repeated (2^nd^ cycle). Then, the root canal was irrigated with 1 mL of 17% EDTA for 30 seconds, and the Clearsonic-R1 insert was used again for 15 seconds (3^rd^ cycle).^11^ Finally, the root canal was irrigated with 5 mL of saline solution, aspirated, and dried with absorbent paper points (Tanari) (Figure 2e).

All of the operative procedures described above were performed by a endodontic specialist with more than five years of clinical experience.

### Stereomicroscope analysis

**Figure 3:**
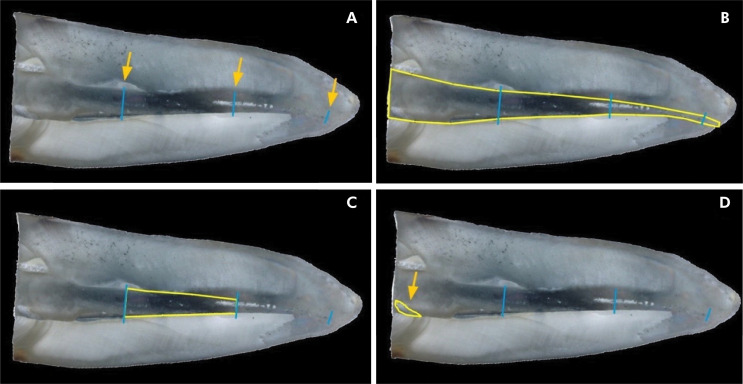
Representative images of the root hemi section in the stereomicroscopy analysis: (a) Root canal hemi section divided into three thirds: cervical, middle, and apical, arrows indicate the root third limits, the last arrow indicates the final millimeter of the root canal; (b) External contour of the total area of the root canal; (c) External contour of the middle third of the root canal; (d) Area containing the remaining filling material attached to the root canal wall (arrow).

Longitudinal grooves were made on each root’s buccal and lingual external surfaces with a double-sided diamond disc No. 7016 (American Burrs). Next, each root was gently cleaved with the aid of a chisel and hammer to avoid the dislodgement of the remaining filling material attached to the root canal walls. Each root hemi section was analyzed in a stereomicroscope (SteREO Discovery. V12, Carl Zeiss, Jena, Germany) at 8× magnification. Image acquisition was performed using the AxioVision software (AxioVision LE64 - SteREO Discovery. V12, Carl Zeiss). A calibrated examiner saved the acquired images in TIF format and individually analyzed them using Image J software (https://imagej.nih.gov/ij/index.html). To calculate the percentage of remaining filling material attached to the root canal walls, the total area of the root canal was delimited (mm^2^) and segmented into three thirds: cervical, middle, and apical. The final 1 mm of the apical third (cemental canal) was also delimited to assess the amount of remaining filling material attached to this portion of the root canal. The outer contour of the different root canal portions and the area of the remaining filling material were outlined using the commands “Analyze>Tools>Grid” and the “Draw line or point grids” plugin of the Image J software.^4,13^ The software was always calibrated using a ruler to ensure a reliable and standard measurement of each specimen. The generated values of the areas (in mm^2^) were transformed into a percentage for comparison among the experimental groups (Figure 3).^4,13^ Both the total area of the root canal and the area of each root third and the final millimeter of the apical portion were considered as reference for calculating the percentage of remaining filling material attached to the root canal walls. The entire analysis was conducted by an examiner who had been previously trained in the software’s features and calibrated to distinguish the remaining filling material from the root canal walls. The images acquired for training and calibration were not included in the subsequent analysis.^4,13^ The analysis was performed blindly.

### Scanning Electron Microscope (SEM) analysis

**Figure 4:**
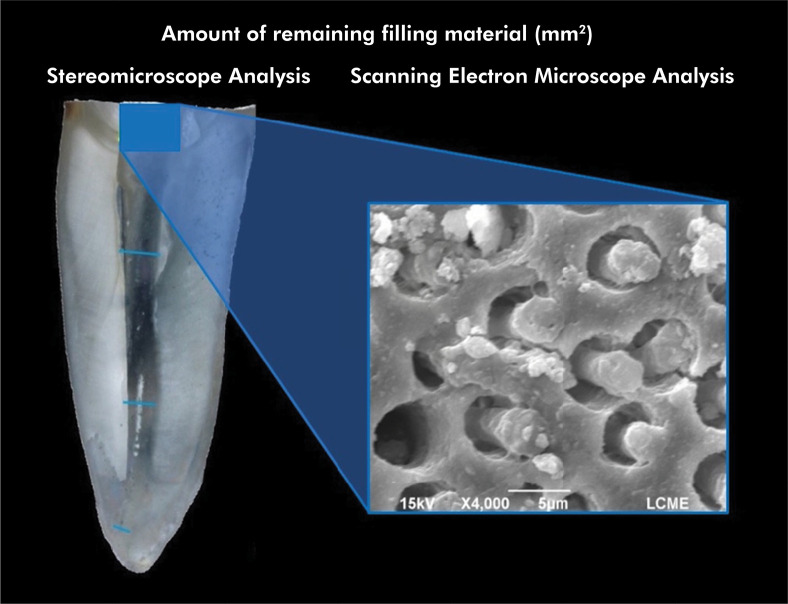
Representative image of the stereomicroscopy and SEM analyses (detail) of the remaining filling material attached to the root canal walls after the use of different supplementary cleaning protocols. The SEM image illustrates the presence of sealer tags inside the dentinal tubules.

To illustrate and complement the assessment performed under a stereomicroscope, representative specimens of each experimental group were analyzed using a JSM-6390LV microscope (JEOL, Tokyo, Japan) at 10 keV. The specimens were air-dried and sputter-coated with a gold/palladium layer (300 Å), and the most representative areas were examined under 500×, 1000×, 2000×, and 4000× magnifications by a previously calibrated examiner (Figure 4). To reduce potential bias, the SEM images underwent a reevaluation one month after the initial measurements were recorded. There were no notable differences between the two assessments, demonstrating strong intra-examiner agreement (Kappa > 0.75). The examiner’s calibration process involved presenting examples of SEM images from the various experimental groups to differentiate the remaining filling material from the root canal walls. The SEM images used for calibration were not included in the later analysis. The SEM analysis was also performed blindly.

### Statistical analysis

The statistical tests were conducted using JAMOVI software (version 1.2) (https://www.jamovi.org), with a significance level set at 5%. The normality of the data was assessed using the Shapiro-Wilk test, and variance homogeneity was examined using the Levene test. As the data exhibited both normality and homogeneity, the initial comparison among experimental groups (amount of remaining filling material), considering the total area of the root canal, was performed using a one-way analysis of variance (ANOVA). Subsequently, a two-way ANOVA was employed to assess the effect of ultrasonic sealer activation/supplementary protocol and root-thirds, as well as the interaction between these factors, on the amount of remaining filling material. Post hoc pairwise comparisons were conducted using the Tukey test for both analyses. The SEM images were analyzed qualitatively.

## Results

### Stereomicroscope analysis

No specimen was lost during the experiment. There was no differentiation between gutta-percha and endodontic sealer during the analysis. Regardless of the supplementary protocol used during endodontic reintervention, the filling material could not be completely removed in any of the specimens (Tables 1 and 2). The different experimental conditions (sealer activation and supplementary removal protocol) had a significant effect on the amount of remaining filling material, and the interaction of these two factors was significant (p < 0.05).

**Table 1:** Mean values (%) and standard deviation of the amount of remaining filling material considering the total area of the root canal.

Groups	Remaining filling material (%)
NoUA	31.4 (8.7)^AB^
NoUA+XP	35.4 (9.9)^BC^
NoUA+CS	23.1 (6.4)^A^
UA	30.3 (8.9)^AB^
UA+XP	45.1 (8.9)^C^
UA+CS	39.6 (11.6)^BC^

Different uppercase letters indicate statistical differences among experimental groups. One-way ANOVA and Tukey’s post hoc test (p < 0.05). NoUA: no ultrasonic activation of the sealer; NoUA+XP: no ultrasonic activation of the sealer + XP-endo Finisher; NoUA+CS: no ultrasonic activation of the sealer + Clearsonic-R1; UA: ultrasonic activation of the sealer; UA+XP: ultrasonic activation of the sealer + XP-endo Finisher; and UA+CS: ultrasonic activation of the sealer + Clearsonic-R1.

**Table 2:** Mean values (%) and standard deviation of the amount of remaining filling material considering each root canal third individually (cervical, medium, and apical) and the final millimeter of the apical portion.

Groups	Root canal thirds			
	Cervical	Middle	Apical	Final apical millimeter
NoUA	30.8 (8.5)^AB,a^	37.9 (11.1)^A,a^	45.5 (12.4)^A,a^	35.1 (13.8)^A,a^
NoUA+XP	34.1 (6.6)^AB,a^	39.9 (12.5)^A,a^	46.2 (13.3)^A,a^	48.2 (13.4)^ABC,a^
NoUA+CS	21.7 (4.4)^A,a^	27.2 (6.3)^A,ab^	39.5 (9.3)^A,b^	61.9 (9.3)^BC,c^
UA	29.6 (8.7)^AB,a^	38.7 (12.3)^A,a^	39.5 (10.2)^A,a^	44.2 (13.8)^A,a^
UA+XP	44.0 (9.1)^B,a^	41.2 (10.6)^A,a^	54.3 (16.2)^AB,a^	46.2 (12.3)^AB,a^
UA+CS	23.8 (6.8)^A,a^	40.2 (7.4)^A,a^	66.8 (5.3)^B,b^	65.0 (9.5)^C,b^

Different uppercase letters indicate a statistical difference in the columns, and different lowercase letters indicate a statistical difference in the lines. Two-way ANOVA and Tukey’s post hoc test (p < 0.05). NoUA: no ultrasonic activation of the sealer; NoUA+XP: no ultrasonic activation of the sealer + XP-endo Finisher; NoUA+CS: no ultrasonic activation of the sealer + Clearsonic-R1; UA: ultrasonic activation of the sealer; UA+XP: ultrasonic activation of the sealer + XP-endo Finisher; and UA+CS: ultrasonic activation of the sealer + Clearsonic-R1.

The NoUA+CS group had the lowest amount of remaining filling material attached to the root canal walls, and it was statistically different from the NoUA+XP (p = 0.046), UA+XP (p < 0.001), and UA+CS (p = 0.003) groups. No statistical differences were found in comparison with the NoUA (p = 0.355) and UA (p = 0.512) groups. The groups without supplementary protocol (NoUA and UA) differed statistically from the UA+XP group (p < 0.019).

The interaction between sealer activation/supplementary removal protocol and root thirds was significant (p < 0.001). No intragroup statistical difference was found concerning the root canal thirds for the NoUA, NoUA+XP, UA, and UA+XP groups (p > 0.05). In the NoUA+CS group, the cervical third had a statistical difference from the apical third and the final millimeter of the apical portion (p < 0.039). However, it was not different from the middle third (p = 1.000). The middle and apical thirds had a statistical difference compared to the final millimeter of the apical portion (p < 0.001). However, they were not different from each other (p = 0.596). In the UA+CS group, the cervical and middle thirds had a statistical difference compared to the apical third and final millimeter of the apical portion (p < 0.001).

No statistical difference was found among the experimental groups in the middle third (p > 0.05). In the cervical third, the NoUA+CS and UA+CS groups had a statistical difference compared to the UA+XP group (p < 0.006). In the apical third, the NoUA, NoUA+XP, UA, and NoUA+CS groups had a statistical difference compared to the UA+CS group (p < 0.004). As for the final millimeter of the apical portion, the NoUA and UA groups had a statistical difference compared to the NoUA+CS and UA+CS groups (p < 0.043). The UA+XP group had a statistical difference compared to the UA+CS group (p = 0.019).

### SEM analysis

**Figure 5:**
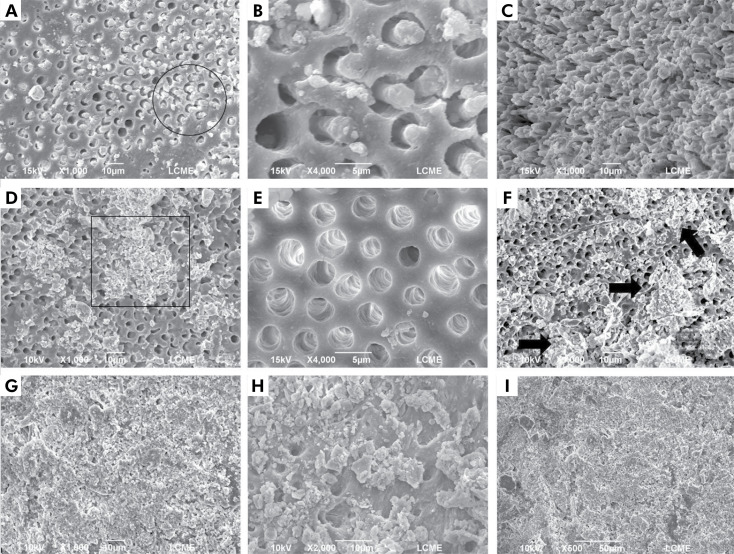
Representative SEM images of specimens from the different experimental groups: (a) Specimen from the UA+XP group: sealer tags within the dentinal tubules (circle); (b) Close-up view (4000×) of the remaining sealer tags in the dentinal tubules; (c) Specimen from the UA+CS group: sealer tags in greater number and density after ultrasonic activation (apical third); (d) Remaining filling material (box) attached to the root canal walls; (e) Specimen from the NoUA+CS group: dentin substrate free of filling material (cervical third); (f) Specimen from the UA+CS group: most of the final apical millimeter is still covered by the remaining filling material (arrows); (g) Specimen from the UA group: remaining filling material is attached to the root canal walls despite re-instrumentation; (h) Close-up view (2000×); (i) Specimen from the UA+XP group: the presence of debris produced by re-instrumentation are seen covering the root canal walls in the apical third and obliterating the entrance of the dentinal tubules.

Representative SEM images are shown in Figure 5. The SEM analysis corroborated the findings observed in the stereomicroscope analysis. The presence of remaining filling material attached to the root canal walls was noticeable in the different root thirds, including the final millimeter of the apical portion. This observation was consistent across all the experimental groups, regardless of the sealer activation or the supplementary protocol employed. A larger amount of filling material, covering most of the root canal walls, was noticed in the apical third and in the last millimeter of the apical portion, as opposed to the cervical and middle thirds.

Similar to the stereomicroscope analysis, the gutta-percha and endodontic sealer could not be distinguished from each other. The mechanical action of the instruments used during filling material removal resulted in a uniformly colored mass. Nevertheless, in SEM, sealer tags could be observed in the dentinal tubules at different areas of the root canal.

Furthermore, scattered amounts of debris produced during root canal re-instrumentation were also noted in these areas, especially in the XP-endo Finisher groups.

## Discussion

The purpose of the present *in vitro* study was to evaluate the effect of ultrasonic activation of the endodontic sealer on the amount of remaining filling material after the application of different supplementary removal protocols during endodontic reintervention. Based on the results obtained, the three null hypotheses tested were rejected, as the ultrasonic activation of the sealer, the supplementary removal protocol, and the root canal level affected the amount of filling material attached to the root canal walls. Furthermore, the UA+XP and UA+CS groups had a greater amount of remaining filling material than the NoUA group, in which no ultrasonic activation or use of supplementary protocols was performed.

Ultrasonic activation promotes greater penetration of the sealer into the dentinal tubules and lateral canals,^2^ resulting in a more homogeneous interface between the filling material and intraradicular dentin^3^ and consequently greater bond strength.^3^ The acoustic microstreaming energy produced by the ultrasonic insert drives the sealer against the root canal walls, reaching anatomically complex areas of difficult access.^1-3,13^ Furthermore, the increase in temperature caused by the activation reduces the viscosity of the sealer, favoring its flow into gaps and voids.^1,3,13,14^ In the present study, the ultrasonic activation of the sealer was performed, based on the positive literature on this subject.^1-4,13^

The epoxy resin-based sealer (AH Plus Jet) is considered the gold standard endodontic sealer because of its outstanding physicochemical and biological characteristics.^15^ Studies have shown that the ultrasonic activation of AH Plus increases its bond strength to intraradicular dentin.^3^ However, even without ultrasonic activation, AH Plus has an excellent bond strength to intraradicular dentin due to its ability to chemically interact with the dentinal network of collagen fibrils, forming covalent bonds between the rings of its epoxy group and the exposed collagen amines groups.^3,16^ Using 17% EDTA before root canal obturation is pivotal in promoting demineralization of the dentin substrate and, consequently, exposure of the collagen fibril network.^4^

Proper penetration of the endodontic sealer into the dentinal tubules is also crucial as it traps residual bacteria that were not eliminated during the root canal preparation.^14^ However, if endodontic reintervention is necessary, it is essential to completely remove the filling material^7^to avoid a persistent infection caused by the bacterial content buried inside the dentinal tubules.^17^

Conversely, many studies have reported that it is impossible to completely remove the filling material during endodontic reintervention.^6,7^ This fact was also observed in the present study since no supplementary protocol tested could eradicate the filling material. da Silva Machado et al.^4^(2021) has also demonstrated that the ultrasonic activation of the sealer may hinder its removal from the root canal walls, leading to a higher percentage of remaining filling material. However, in the study mentioned above, no supplementary removal protocol was adopted during endodontic reintervention.

Several instruments and techniques may be applied for filling material removal, ranging from sonic and ultrasonic instruments^18,19^to laser devices.^20^ This study used the XP-endo Finisher rotary instrument and the Clearsonic-R1 ultrasonic insert as additional steps during endodontic reintervention. The choice of these instruments was based on the scientific literature, which shows a significant reduction in the amount of remaining filling material attached to the root canal walls after their use.^8-11,21^ There are two versions of the XP-endo instrument, the XP-endo Finisher and the XP-endo Finisher R.^8,10^ The XP-endo Finisher R was specially developed to optimize root canal cleaning during endodontic reintervention.^8,10^ According to Silva et al.^8^(2018), both instruments had similar performance, with no significant difference in the amount of remaining filling material after root canal re-instrumentation. Therefore, the XP-endo Finisher version was used in this study.

Compared to ultrasonic inserts, such as the Clearsonic-R1, studies have shown that the XP-endo Finisher instrument enables better filling material removal.^10,11^ Conversely, in the present study, when comparing the NoUA+XP and NoUA+CS groups, a lower amount of remaining filling material was noted when the ultrasonic insert was used. Furthermore, when comparing the same instruments in groups where the sealer was ultrasonically activated, a smaller amount of material was observed for the Clearsonic group. Although the differences were not statistically significant, it is a relevant clinical finding.

The differences observed between the two supplementary protocols may be explained by their different mode of action and behavior in root canals with an oval cross-section.^11,19^ The protocols of the two instruments were based on the study by Tavares et al.^11^However, for the XP-endo Finisher instrument, the irrigating solutions (2.5% NaOCl solution and 17% EDTA) were previously heated, according to the methodology by Silva et al.^8^ The heating of the solutions was intended to simulate body temperature and promote the phase transformation (martensitic-austenitic) of the alloy from which the instrument is made. Therefore, it was possible to change the instrument’s shape (“sickle”) to achieve its best performance.

The results obtained in the present study agree with previous studies that reported that even after using the additional step with the XP-endo Finisher instrument or the Clearsonic-R1 insert, remnants of filling material persist.^8^ Furthermore, Machado et al.^4^reported that ultrasonic activation of the endodontic sealer hinders the removal of the material. However, in the present study, statistical difference was found only in one of the activated groups compared to the other experimental groups.

Within the groups where the sealer was ultrasonically activated, there was a statistical difference between the supplementary protocols used. A greater amount of remaining filling material was noted in the XP-endo Finisher group in comparison with the Clearsonic-R1 group. However, the Clearsonic-R1 group did not differ from the group with traditional filling material removal.

Using the XP-endo Finisher instrument as an additional step did not reduce the amount of filling material residue compared to the groups that used only the R40 and R50 instruments for filling removal and re-instrumentation, respectively. This finding disagrees with those of Silva et al.,^8^ which showed a significant reduction in the amount of filling material after using the XP-endo Finisher instrument. The method of analysis may be the reason for the discrepant results. In the present study, the analysis was performed under a stereomicroscope. In contrast, in the study by Silva et al.,^8^ a micro-CT analysis was performed. Many studies have used micro-CT scanners to quantify the remaining filling material after endodontic reintervention.^8-11^ However, micro-CT has a high cost, which restricts access. In addition, it takes a long time to scan the specimens and artifacts could be observed in the presence of radiodense materials, such as sealer and gutta-percha.^22,23^ Despite the limitations of a 2D method, the stereomicroscope is still widely used.^4,13,22,23^ It enables direct observation of the specimen without the distortions and artifacts that can occur with radiographic and tomographic methods.^22^ Despite the advances in software designed to mitigate artifacts in tomographic images, radiodense materials can still cause distortions that complicate the analysis of certain anatomical regions.^22^

In the present study, SEM analysis was adopted to complement the assessment of stereomicroscopy, which allowed the visualization of sealer tags inside the dentinal tubules and even in areas that seemed to be clear. Although micro-CT analysis may be considered a “gold standard” method, it is not possible to evaluate the presence of filling material remnants inside the dentinal tubules.^23^ Therefore, the use of additional complementary methods, such as SEM, is crucial to ensure a reliable analysis.

The groups in which the additional step was applied had a more significant amount of remaining filling material, except for the NoUA+CS group. Therefore, the filling material removal and the re-instrumentation of the root canal with reciprocating instruments with a tip diameter of 0.40 mm (R40) and 0.50 mm (R50) before the use of the XP-endo Finisher instrument, which has a tip diameter of 0.25 mm, may have negatively affected its performance, making it ineffective in these cases.^8^

It is important to note that the removal of filling material may be impeded by the shape of the root canal.^8,10,11,24^ In this study, lower and upper canine teeth with similar anatomical characteristics were selected to standardize the sample. Canine teeth typically have wide and straight root canals; however, their oval cross-section may present an additional challenge for removing filling material, especially at the apical portion.^8,25^The root canal is narrow mesiodistally and wide buccolingually.^24^ Comparatively, the lingual root canal wall exhibits a slit-like shape compared to the larger buccal root canal wall, which can impede shaping and cleaning efforts.^24^ On the other hand, it is important to note that the results cannot be extrapolated to teeth with greater anatomical complexity, such as posterior teeth. Furthermore, the use of the ultrasonic insert close to the working length, as performed in this study, may not be feasible in teeth with curved roots, which are among the most challenging in clinical practice.^9^

As the apical portion is a critical area for cleaning due to its peculiar anatomical characteristics,^25^ a specific analysis of the final millimeter of the apical third was performed. However, the different supplementary protocols had similar performance concerning the amount of remaining filling material in this region. SEM analysis revealed that the apical region had more significant amounts of remaining filling material than the other root thirds, which may be explained by the difficulties imposed by the region’s anatomy.^14^ Alcalde et al.^14^have reported that ultrasonic activation of AH Plus sealer increases its intratubular penetration, especially in the apical third. In addition, the irrigation in this area is highly compromised due to the difficulty if reaching this region with needles and instruments, hindering the removal of the filling material and debris,^14^ as observed by SEM.

This study’s relevance and clinical applicability are worth mentioning since the ultrasonic activation of the endodontic sealer increases its penetration within the dentinal tubules, isthmus regions, and accessory canals, hindering filling material removal when endodontic reintervention is necessary.^4^ Conversely, the activation of the root canal sealer should never be presented as a disadvantage. Any endodontic treatment should apply the best available technique for successful results. Therefore, professionals should be aware of supplementary protocols that are effective in removing the filling material in cases where the sealer was ultrasonically activated. The results of the present study bridged a gap in the current literature. However, further studies assessing different supplementary removal protocols in root canals obturated after ultrasonic sealer activation should be carried out.

## Conclusions

XP-endo Finisher instrument demonstrated the least effective performance when used as an additional step following the ultrasonic activation of the endodontic sealer. Conversely, the Clearsonic-R1 insert exhibited the most favorable performance when the sealer was not subjected to ultrasonic activation.

## References

[1] Guimarães BM, Amoroso-Silva PA, Alcalde MP, Marciano MA, Andrade FB, Duarte MA (2014). Influence of ultrasonic activation of 4 root canal sealers on the filling quality. J Endod.

[2] Arslan H, Abbas A, Karatas E (2016). Influence of ultrasonic and sonic activation of epoxy-amine resin-based sealer on penetration of sealer into lateral canals. Clin Oral Investig.

[3] Wiesse PE, Silva-Sousa YT, Pereira RD, Estrela C, Domingues LM, Pécora JD (2018). Effect of ultrasonic and sonic activation of root canal sealers on the push-out bond strength and interfacial adaptation to root canal dentine. Int Endod J.

[4] Machado APS, Souza ACCC, Gonçalves TL, Marques AAF, Garcia LFR, Bortoluzzi EA (2021). Does the ultrasonic activation of sealer hinder the root canal retreatment?. Clin Oral Investig.

[5] Esposito M, Trullenque-Eriksson A, Tallarico M (2018). Endodontic retreatment versus dental implants of teeth with an uncertain endodontic prognosis: 3-year results from a randomised controlled trial. Eur J Oral Implantology.

[6] Rossi-Fedele G, Ahmed HM (2017). Assessment of root canal filling removal effectiveness using micro-computed tomography: a systematic review. J Endod.

[7] Rosa AF, Fischer BV, Dias-Junior LC, Serique AV, Bortoluzzi EA, Teixeira CD (2024). Effectiveness of different supplementary protocols for remaining filling material removal in endodontic reintervention: an integrative review. Odontology.

[8] Silva EJ, Belladonna FG, Zuolo AS, Rodrigues E, Ehrhardt IC, Souza EM (2018). Effectiveness of XP-endo Finisher and XP-endo Finisher R in removing root filling remnants: a micro-CT study. Int Endod J.

[9] Aksel H, Küçükkaya Eren S, Askerbeyli Örs S, Serper A, Ocak M, Çelik HH (2019). Micro-CT evaluation of the removal of root fillings using the ProTaper Universal Retreatment system supplemented by the XP-Endo Finisher file. Int Endod J.

[10] De-Deus G, Belladonna FG, Zuolo AS, Cavalcante DM, Carvalhal JC, Simões-Carvalho M (2019). XP-endo Finisher R instrument optimizes the removal of root filling remnants in oval-shaped canals. Int Endod J.

[11] Tavares SJ, Gomes CC, Marceliano-Alves MF, Guimarães LC, Provenzano JC, Amoroso-Silva P (2021). Supplementing filling material removal with XP-Endo Finisher R or R1-Clearsonic ultrasonic insert during retreatment of oval canals from contralateral teeth. Aust Endod J.

[12] Nagendrababu V, Murray PE, Ordinola-Zapata R, Peters OA, Rôças IN, Siqueira JF (2021). PRILE 2021 guidelines for reporting laboratory studies in Endodontology: A consensus-based development. Int Endod J.

[13] Fischer BV, Dias-Junior LC, Minamisako MC, Almeida CM, Silva LR, Bortoluzzi EA (2024). Effect of the timing of primary endodontic treatment and dosage of radiation therapy on the filling material removal. Aust Endod J.

[14] Alcalde MP, Bramante CM, Vivan RR, Amorso-Silva PA, Andrade FB, Duarte MA (2017). Intradentinal antimicrobial action and filling quality promoted by ultrasonic agitation of epoxy resin-based sealer in endodontic obturation. J Appl Oral Sci.

[15] Marciano MA, Guimarães BM, Ordinola-Zapata R, Bramante CM, Cavenago BC, Garcia RB (2011). Physical properties and interfacial adaptation of three epoxy resin-based sealers. J Endod.

[16] Sousa-Neto MD, Coelho FIS, Marchesan MA, Alfredo E, Silva-Sousa YT (2005). Ex vivo study of the adhesion of an epoxy-based sealer to human dentine submitted to irradiation with Er: YAG and Nd: YAG lasers. Int Endod J.

[17] Haapasalo M, Shen YA, Ricucci D (2008). Reasons for persistent and emerging post-treatment endodontic disease. Endod Topics.

[18] Martins MP, Duarte MA, Cavenago BC, Kato AS, Bueno CES (2017). Effectiveness of the protaper next and reciproc systems in removing root canal filling material with sonic or ultrasonic irrigation: a micro-computed tomographic study. J Endod.

[19] Rivera-Peña ME, Duarte MA, Alcalde MP, Andrade FB, Vivan RR (2018). A novel ultrasonic tip for removal of filling material in flattened/oval-shaped root canals: a microCT study. Braz Oral Res.

[20] Keles A, Arslan H, Kamalak A, Akçay M, Sousa-Neto MD, Versiani MA (2015). Removal of filling materials from oval-shaped canals using laser irradiation: a micro-computed tomographic study. J Endod.

[21] Campello AF, Almeida BM, Franzoni MA, Alves FR, Marceliano-Alves MF, Rôças IN (2019). Influence of solvent and a supplementary step with a finishing instrument on filling material removal from canals connected by an isthmus. Int Endod J.

[22] Nair MK, Nair UP (2007). Digital and advanced imaging in endodontics: a review. J Endod.

[23] Luiz CM, Goulart TS, Magalhães KS, Souza GR, Garcia LD, Almeida J (2024). Do ultraconservative access cavities hinder endodontic reintervention in mandibular incisors? A laboratory investigation. Int J Dent.

[24] Versiani MA, Leoni GB, Steier L, De-Deus G, Tassani S, Pécora JD (2013). Micro-computed tomography study of oval-shaped canals prepared with the self-adjusting file, Reciproc, WaveOne, and ProTaper universal systems. J Endod.

[25] Mjör IA, Smith MR, Ferrari M, Mannocci F (2001). The structure of dentine in the apical region of human teeth. Int Endod J.

